# Longer leukocyte telomere length increases cardiovascular mortality in type 2 diabetes patients

**DOI:** 10.1111/1753-0407.13376

**Published:** 2023-03-08

**Authors:** Ziwei Chen, Yao Shen, Jing He, Yang Shen, Weida Zhu, Xiaohui Wu, Mingbing Xiao

**Affiliations:** ^1^ Department of Cardiology Affiliated Hospital of Nantong University Nantong China; ^2^ Research Center of Clinical Medicine Affiliated Hospital of Nantong University Nantong China; ^3^ Eye Institute Affiliated Hospital of Nantong University Nantong China; ^4^ Department of Chemotherapy Affiliated Hospital of Nantong University Nantong China

**Keywords:** cross‐sectional study, diabetes, leukocyte telomere length, mortality, NHANES, 横断面研究, 糖尿病, 白细胞端粒长度, 死亡率, NHANES

## Abstract

**Aims:**

Leukocyte telomere length (LTL), as a biomarker of biological aging, is associated with the prevalence and complications of diabetes. This study aims to investigate the associations between LTL and all‐cause and cause‐specific mortality in patients with type 2 diabetes.

**Methods:**

All participants with baseline LTL records were included from the National Health and Nutrition Examination Survey 1999–2002. Death status and its causes were ascertained for National Death Index based on *International Classification of Diseases, Tenth Revision* code. Cox proportional hazards regression models were established to estimate the hazard ratios (HRs) of LTL associating with all‐cause and cause‐specific mortality.

**Results:**

The study enrolled 804 diabetic patients with the mean follow‐up of 14.9 ± 2.59 years. There were 367 (45.6%) all‐cause deaths, 80 (10.0%) cardiovascular deaths, and 42 (5.2%) cancer‐related deaths. Longer LTL was associated with reduced all‐cause mortality, whereas this association disappeared after adjusting for other variables. Compared with the lowest tertiles of LTL, the multivariable‐adjusted hazard ratio of cardiovascular mortality was 2.11 (95% confidence interval [CI] 1.31–3.39; *p* < .05) in the highest tertiles. In terms of cancer mortality, the highest tertile was negatively correlated with the risk of cancer mortality (HR 0.58 [95% CI 0.37, 0.91], *p* < .05).

**Conclusion:**

In conclusion, LTL was independently associated with the risk of cardiovascular mortality in patients with type 2 diabetes and was negatively correlated with the risk of cancer mortality. Telomere length may be a predictor of cardiovascular mortality in diabetes.

## Graphical abstract

**Highlights**

- Leukocyte telomere length was independently associated with the risk of cardiovascular mortality.
- Leukocyte telomere length was not associated with the risk of all‐cause mortality in patients with diabetes.
- Telomere length may be a predictor of cardiovascular mortality in diabetes.

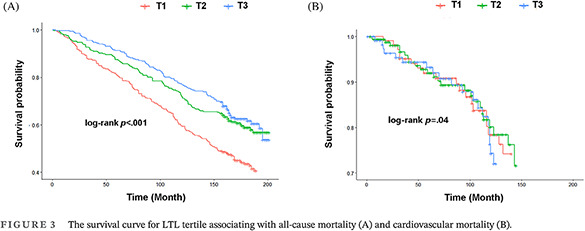

## 摘要

**目的:**白细胞端粒长度(LTL)作为生物学衰老的生物标志物, 与糖尿病的患病率和并发症相关。本研究旨在探讨2型糖尿病患者LTL与全因死亡率、原因特异性死亡率之间的关联。

**方法:**纳入1999‐2002年全国健康与营养调查(NHANES)中有基线LTL记录的研究对象。根据国际疾病分类10(ICD‐10)编码, 使用国家死亡索引(National Death Index)对研究对象的死亡状况及其死因进行确认。建立Cox比例风险回归模型评估LTL与全因死亡率及原因特异性死亡率的关联风险比。

**结果:**纳入804例糖尿病患者, 平均随访14.9±2.59年。其中全因死亡367例(45.6%), 心血管疾病死亡80例(10.0%), 癌症相关死亡42例(5.2%)。较长的LTL与降低全因死亡率相关, 而在调整其他变量后, 这种相关性消失。与LTL的最低分位数相比, 心血管死亡率的多变量校正风险比和95%可信区间为2.11 (1.31‐3.39;P<0.05)。在癌症死亡率方面, 最高三分位数与癌症死亡风险呈负相关(HR 0.58 [0.37,0.91], P<0.05)。

**结论:**综上所述, LTL与2型糖尿病患者心血管死亡风险独立相关, 与恶性肿瘤死亡风险呈负相关。端粒长度可能是糖尿病患者心血管死亡率的预测因子。

## 1 INTRODUCTION

Diabetes is a leading cause of morbidity and mortality worldwide.^1^, ^2^ Patients with diabetes have 2 ~ 4 times risk to develop cardiovascular diseases (CVDs).^3^ An international registry found that the risk of cardiovascular death was greater in patients with diabetes compared with nondiabetes (16.5% versus 13.1%; adjusted hazard ratio [HR] 1.27).^4^ In addition, diabetes was associated with a greater risk of heart failure, nonfatal myocardial infarction, and stroke.^5^ Besides, the diabetes status was involved in aging, oxidative stress, and chronic inflammation, promoting shortening of telomeres.

Telomeres are the TTAGGG repetitive DNA sequences at the ends of eukaryotic chromosomes and are responsible for maintaining the genome integrity during cell division.^6^ Excess oxidative stress and proinflammatory mediators like tumor necrosis factor‐alpha, interferon‐gamma, interleukin‐6 and interleukin‐10 were two mechanisms contributing to age‐dependent leukocyte telomere length (LTL) shortening.^7^, ^8^ Highly shortened telomeres can cause cellular senescence and subsequent arrest in the cell cycle.^9^ Epidemiological studies have shown that shorter LTL was associated with a number of age‐related disorders such as CVDs, neurodegenerative diseases, and cancers.^10^, ^11^, ^12^ A community‐based cohort study found that shorter LTL was associated with increased overall cardiovascular, respiratory, digestive, and musculoskeletal mortality but not cancer‐related mortality.^13^ The telomere system played an important role in the pathogenesis and progression of diabetes mellitus as well as in its vascular complications.^14^ However, the prognostic effect of telomere length on cardiovascular mortality or cancer mortality remains unclear in diabetes patients.

Hence, this study was designed to evaluate the relationship between LTL and all‐cause and cause‐specific mortality in type 2 diabetes by using data from the National Health and Nutrition Examination Survey (NHANES).

## 2 METHODS

### 2.1 Study population

**FIGURE 1:**
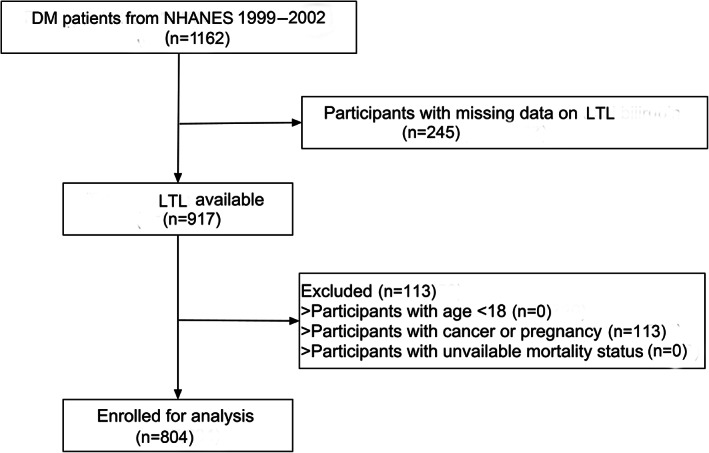
The flow chart of participants selection. DM, diabetes mellitus; LTL, leukocyte telomere length; NHANES, National Health and Nutrition Examination Survey.

Our study included individuals from the NHANES. A total of 1162 patients with type 2 diabetes were included from NHANES cycle of 1999–2002. After excluding those with missing LTL records (*n* = 245) and with cancer or pregnancy (*n* = 113), a total of 804 participants were enrolled in our study. Figure 1 depicted the selection process. The protocol was approved by the Ethics Review Board of National Center for Health Statistics (Protocol # 98–12).

### 2.2 Leukocyte telomere length measurement

Genomic DNA was extracted from the buffy coat in the peripheral blood sample using QIAamp 96 DNA Blood Kits (Qiagen, Valencia, CA) according to the manufacturer's protocol. Relative telomere length was measured by comparing the ratio of telomere repeat number (T) to a single copy gene number for albumin (S) in the experimental sample to a standardized reference sample value using multiplexed quantitative polymerase chain reaction method. All experimental samples were assayed in duplicate, and the average value of the two replicates was used for the final analysis for each subject. The mean of the T/S ratio values was calculated, and the largest and the smallest T/S ratio values were marked as potential outliers. If the absolute value of the log of the ratio between the recalculated mean (excluding the potential outliers) to the value of the potential outlier was >0.4, then the value was marked as an outlier. The interassay coefficient of variability for LTL was 4%.

### 2.3 Covariates collection

Information on sex, age, race, education level, smoking status, alcohol habit, prior comorbid illness, and medication use were obtained from standardized questionnaires. Body mass index (BMI, kg/m2) was calculated as weight divided by height squared. Race was classified as non‐Hispanic white, non‐Hispanic black, Mexican American, or other. Education levels were categorized into less than high school, high school or equivalent, and college or above. Family income‐to‐poverty ratio was classified as 0–1.0, 1.0–3.0, or >3.0, indicating a low, middle, and high income status, respectively. Smoking habits were defined as current, past and never. Previous history of diseases (hypertension and CVD) and medications (aspirin, hypoglycemic drugs, and lipid‐lowering drugs) were determined from self‐reported questionnaires.

### 2.4 End points

The primary outcome was all‐cause mortality and secondary outcomes included death from CVD, diabetes, or malignant neoplasms. Mortality status was obtained by linkage to the National Death Index by 31 December 2015. Causes of death included malignant tumors, CVDs, respiratory diseases, Alzheimer's disease, diabetes, nephropathy‐related diseases, accidental death, and other causes. CVDs were defined as *International Classification of Diseases, Tenth Revision* (ICD‐10) codes I00‐I09, I11, I13, or I20‐I51. Malignant neoplasm was defined as ICD‐10 codes C00‐C97 (Supplementary Table S1).

### 2.5 Statistical analysis

**FIGURE 2:**
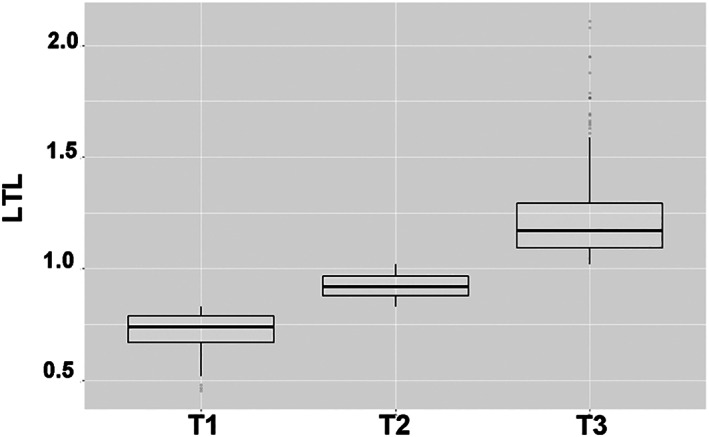
The boxplot of leukocyte telomere length. BMI, body mass index; CI, confidence interval; HR, hazard ratio; LDL‐C, low‐density lipoprotein cholesterol; LTL, leukocyte telomere length; National Health and Nutrition Examination Survey.

Descriptive statistics were presented according to the levels of LTL (T1: <0.83, T2: 0.83–1.02, T3: >1.02; Figure 2) and group differences were explored by one‐way analysis of variance and chi‐square tests. First, we explored the association of LTL tertiles with overall survival using Kaplan–Meier analysis. Associations between levels of LTL and the risk of all‐cause mortality was estimated by classical Cox regression models. We fitted Cox model by specifying the event of interest, and by censoring for competing events. Multiple imputation based on predictive mean matching was performed for covariates with missing values using package *mice* form R (Supplementary Table S2). All analysis were performed using R version 3.6.

## 3 RESULTS

This study included 804 participants with 14.9 ± 2.59 year of follow‐up. The baseline characteristics of the study population according to LTL tertiles (T1: <0.83, T2: 0.83–1.02, T3: >1.02) were shown in the Table 1. Compared with the lowest group, participants in the highest group were more likely to be younger, non‐Hispanic black, and have higher BMI.

**TABLE 1:** Characteristics of the study population according to the leukocyte telomere length.

Variable	All (*n* = 804)	T1 (*n* = 268)	T2 (*n* = 269)	T3 (*n* = 267)	*p* value
LTL	0.96 (0.11)	0.73 (0.08)	0.92 (0.05)	1.23 (0.19)	<.001
Male sex (%)	435 (54.1)	143 (53.4)	151 (56.1)	141 (52.8)	.709
Age, years	61.3 (13.9)	67.0 (11.9)	61.4 (12.4)	55.4 (14.80)	<.001
Race (%)					
Non‐Hispanic white	294 (36.6)	119 (44.4)	97 (36.1)	78 (29.2)	.001
Non‐Hispanic black	193 (24.0)	48 (17.9)	67 (24.9)	78 (29.2)	
Mexican American	240 (29.9)	85 (31.7)	78 (29.0)	77 (28.8)	
Others	77 (9.6)	16 (6.0)	27 (10.0)	34 (12.7)	
Education (%)					.283
Less than high school	416 (51.7)	152 (56.7)	134 (49.8)	130 (48.7)	
High school or equivalent	161 (20.0)	52 (19.4)	52 (19.4)	57 (21.3)	
College or above	227 (28.2)	64 (23.9)	83 (31.0)	80 (30.0)	
BMI, kg/m2	31.5 (6.8)	30.4 (6.1)	32.0 (7.1)	32.2 (7.3)	.003
IPR (%)					.01
<1.0	129 (16.1)	116 (13.8)	113 (14.9)	95 (12.2)	
1.0–3.0	361 (45.2)	356 (42.3)	283 (37.2)	298 (38.3)	
>3.0	309 (38.7)	369 (43.9)	364 (47.9)	385 (49.5)	
Smoking (%)					.515
Current	186 (23.1)	60 (22.4)	61 (22.7)	65 (24.3)	
Past	55 (6.8)	13 (4.9)	20 (7.4)	22 (8.2)	
Never	563 (70.0)	195 (72.8)	188 (69.9)	180 (67.4)	
Activity (%)					
Vigorous	296 (36.8)	74 (27.6)	117 (43.5)	105 (39.3)	.002
Moderate	437 (54.4)	168 (62.7)	133 (49.4)	136 (50.9)	
Inactive	71 (8.8)	26 (9.7)	19 (7.1)	26 (9.7)	
Past history (%)					
Hypertension	332 (41.3)	124 (46.3)	102 (37.9)	106 (39.7)	.118
CVD	161 (20.0)	64 (23.9)	54 (20.1)	43 (16.1)	.080
Prior medication (%)					
Aspirin drug	527 (65.5)	174 (64.9)	169 (62.8)	184 (68.9)	.322
Hypoglycemic drug	520 (64.7)	183 (68.3)	159 (59.1)	178 (66.7)	.06
Lipid‐lowering drug	570 (70.9)	205 (76.5)	195 (72.5)	170 (63.7)	.004
LDL‐C, mg/dL	119.4 (33.7)	112.7 (29.6)	126.6 (35.1)	119.0 (34.8)	<.001

*Note*: Data are presented as mean (SD) or *n* (%). T1: <0.83, T2: 0.83–1.02, T3: >1.02.

Abbreviations: BMI, body mass index; CVD, cardiovascular diseases; IPR, income‐to‐poverty ratio; LDL‐C, low‐density lipoprotein cholesterol; LTL, leukocyte telomere length.

**FIGURE 3:**
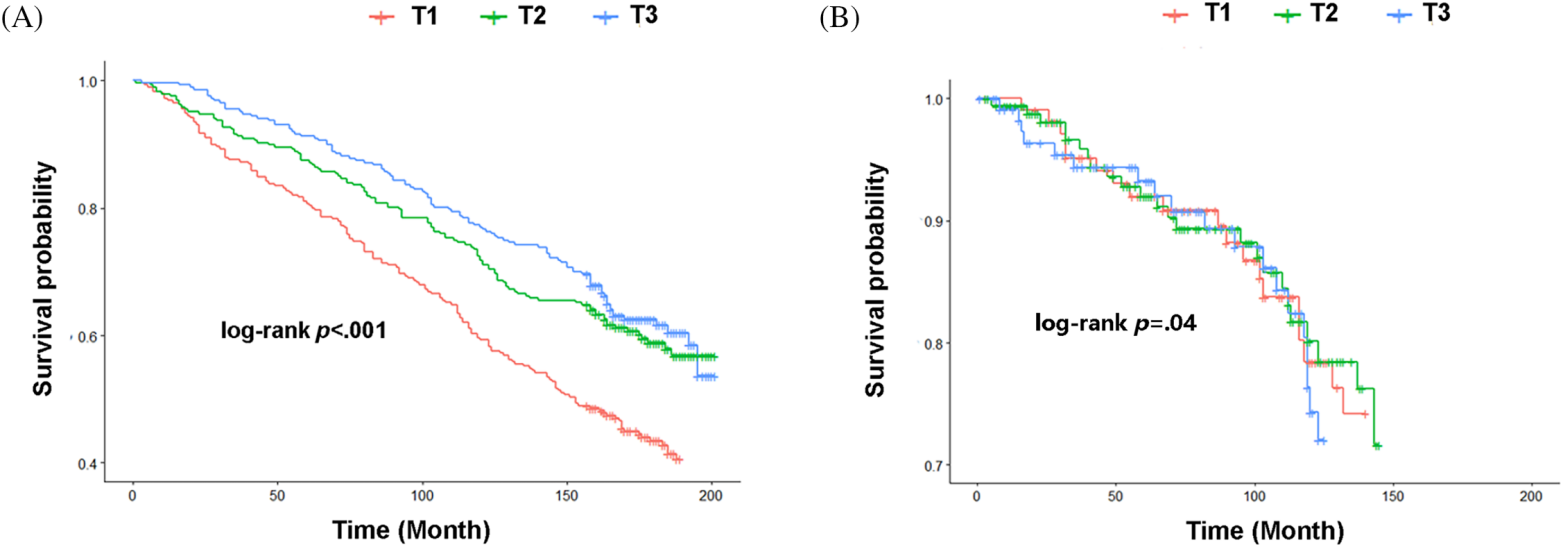
The survival curve for LTL tertile associating with all‐cause mortality (A) and cardiovascular mortality (B).

A total of 367 deaths occurred among 804 participants (45.6%). Kaplan–Meier survival curve showed lower LTL was associated with increased all‐cause mortality (*p* < .001; Figure 3A) and cardiovascular mortality (*p* = .04; Figure 3B).

As shown in Table 2, compared with the lowest tertile, the HR for all‐cause mortality was 0.72 (95% confidence interval [CI] [0.56, 0.93], *p* < 0.05) for individuals with the highest tertile in an adjusted model. However, the significance disappeared after adjusting for gender, age, race, education, BMI, smoking, activity, hypertension, CVD, aspirin, hypoglycemic drugs, lipid‐lowering drugs, and low‐density lipoprotein cholesterol (LDL‐C). Surprisingly, the highest LTL group increased the risk of cardiovascular mortality (HR 1.62 [95% CI 1.02, 1.72], *p* < .05) in an unadjusted model, even in a fully adjusted model. (HR 2.11 [95% CI 1.31, 3.39], *p* < .05). In terms of cancer mortality, the highest tertile was negatively correlated with the risk of cancer mortality (HR 0.58 [95% CI 0.37, 0.91], *p* < .05) after adjusting for gender, age, race, education, BMI, smoking, activity, hypertension, CVD, aspirin, hypoglycemic drugs, lipid‐lowering drugs, and LDL‐C.

**TABLE 2:** Association of leukocyte telomere length with all‐cause and cause‐specific mortality.

All‐cause and cause‐specific mortality	Cases	N	Model 1	Model 2	Model 3
			HR (95% CI)	HR (95% CI)	HR (95% CI)
All causes					
T1	156	268	**Ref**	**Ref**	**Ref**
T2	109	269	0.69 [0.54, 0.88]**	0.86 [0.67, 1.10]	0.92 [0.70, 1.20]
T3	102	267	0.72 [0.56, 0.93]*	1.20 [0.92, 1.55]	1.15 [0.88, 1.50]
Cardiovascular					
T1	27	268			
T2	26	269	0.99 [0.59, 1.66]	1.33 [0.78, 2.26]	1.40 [0.88, 2.24]
T3	27	267	1.62 [1.02, 1.72]*	1.86 [1.10, 3.17]*	2.11 [1.31, 3.39]*
Malignant neoplasms					
T1	17	268			
T2	13	269	0.84 [0.42, 1.71]	0.85 [0.42, 1.73]	0.92 [0.41, 1.38]
T3	12	267	0.72 [0.35, 1.51]	0.75 [0.36, 1.57]	0.58 [0.37, 0.91]*

*Note*: Model 1 was unadjusted. Model 2 was adjusted for age and gender. Model 3 was adjusted for gender, age, race, education, body mass index, smoking, activity, hypertension, cardiovascular disease, aspirin, hypoglycemic drugs, lipid‐lowering drugs, and low‐density lipoprotein cholesterol.

Abbreviations: HR, hazard ratio; CI, confidence interval.

* *p* < .05;

** *p* < .01.

## 4 DISCUSSION

In our study, we found that longer leukocyte telomere length increased the risk of cardiovascular mortality in patients with type 2 diabetes. However, leukocyte telomere length was not associated with all‐cause mortality in diabetic patients. Telomere length may be a predictor of cardiovascular mortality in diabetes.

Previous study reported that shorter LTL was associated with a higher risk of mortality in the general population, which could be explained by increased systemic inflammation and oxidative stress.^15^ It was understandable that our univariate survival analysis also suggested that lower LTL was associated with increased all‐cause mortality. However, after adjusting many covariates, the association disappeared in our study, which suggested that other factors could mediate their association. In the general population, LTL was negatively^16^ or U‐shaped^17^ associated with the risk of all‐cause mortality. On contrary to the previous studies, we did not’ observe a significant association between LTL and all‐cause mortality in diabetic patients. We speculated that telomeres have already shortened substantially among diabetes subjects,^18^ and given the presence of other major risk factors for death, the independent effect of telomere length on mortality may be reversed. Besides, Chen et al found that shortened relative leukocyte telomere length was associated with an increased risk of all‐cause mortality in type 2 diabetes.^19^ The difference may be due to (a) different population (US population in our study and Asian population in the previous study); (b) a lower level of LTL in our study (~0.96) compared with the previous study (~4.6), which could be attributed to the different methods used for the measurement of LTL; and (c) a high all‐cause mortality in our study (45.6%), although it was not implausible, given the starting ages of the subjects and the long follow‐up time.

Surprisingly, we found that longer LTL increased the risk of cardiovascular mortality in patients with diabetes. Although a report found shortening of telomeres would protect against cardiovascular mortality in elderly men,^20^ our results contrasted with other previous reports that associated shorter telomeres with cardiovascular mortality.^21^, ^22^ Chen et al also found that shortened leukocyte telomere length was associated with prevalent and incident cardiovascular complications in type 2 diabetes.^23^ There may be a competing risk against cardiovascular mortality in diabetic patients and it was stronger in shorter LTL subgroups. The relation should be interpreted with some caution until more studies are designed to explore this association. In consistent with previous reports, we found an inverse correlation between LTL and cancer mortality.

Our study has some limitations. First, some covariates were self‐reported. Second, we used mean LTL measured in leukocyte cells, which are a heterogeneous mix of cells with varying telomere length. It is possible that the association may vary depending on cell type used to measure telomere length. Third, data on LTL was collected only once at baseline, and it was unclear whether longitudinal changes over time could affect the association with mortality. Finally, heart diseases or other variables may not be present at baseline.

## 5 CONCLUSION

We found that leukocyte telomere length was independently associated with the risk of cardiovascular mortality, not all‐cause mortality, in patients with type 2 diabetes. Telomere length may be a predictor of cardiovascular mortality in diabetes.

## AUTHOR CONTRIBUTIONS

Xiao Mingbing and Wu Xiaohui designed the study; Chen Ziwei, Shen Yao, and He Jing wrote the main manuscript; Shen Yao and Zhu Weida prepared the figures and tables.

## FUNDING INFORMATION

None.

## CONFLICT OF INTEREST STATEMENT

All authors have nothing to disclose regarding conflict of interest with respect to this manuscript.

## ETHICS STATEMENT

The protocol was approved by the Ethics Review Board of National Center for Health Statistics (Protocol # 98–12).

## Supporting information


**Table S1.** The ICD‐10 codes used in our study.
**Table S2.** The percentage of missing data.

## Data Availability

The data used to support the findings of this study are available from the corresponding author Mingbing Xiao upon request.

## References

[1] Banovic M , Athithan L , McCann GP . Aortic stenosis and diabetes mellitus: An ominous combination. Diab Vasc Dis Res. 2019;16(4):310‐323. doi:10.1177/1479164118820657 30623669

[2] Glovaci D , Fan W , Wong ND . Epidemiology of diabetes mellitus and cardiovascular disease. Curr Cardiol Rep. 2019;21(4):21. doi:10.1007/s11886-019-1107-y 30828746

[3] Fox CS , Coady S , Sorlie PD , et al. Trends in cardiovascular complications of diabetes. JAMA. 2004;292(20):2495‐2499. doi:10.1001/jama.292.20.2495 15562129

[4] Rawshani A , Rawshani A , Franzen S , et al. Risk factors, mortality, and cardiovascular outcomes in patients with type 2 diabetes. N Engl J Med. 2018;379(7):633‐644. doi:10.1056/NEJMoa1800256 30110583

[5] Cavender MA , Steg PG , Smith SC Jr , et al. Impact of diabetes mellitus on hospitalization for heart failure, cardiovascular events, and death: outcomes at 4 years from the reduction of atherothrombosis for continued health (REACH) registry. Circulation. 2015;132(10):923‐931. doi:10.1161/CIRCULATIONAHA.114.014796 26152709

[6] Sudyka J . Does reproduction shorten Telomeres? Towards integrating individual quality with life‐history strategies in telomere biology. Bioessays. 2019;41(11):e1900095. doi:10.1002/bies.201900095 31577044

[7] Zhou JF , Yan XF , Ruan ZR , et al. Heroin abuse and nitric oxide, oxidation, peroxidation, lipoperoxidation. Biomed Environ Sci. 2000;13(2):131‐139. 11055015

[8] Lustig A , Liu HB , Metter EJ , et al. Telomere shortening, inflammatory cytokines, and anti‐cytomegalovirus antibody follow distinct age‐associated trajectories in humans. Front Immunol. 2017;8:1027. doi:10.3389/fimmu.2017.01027 28970831 PMC5609584

[9] Munoz‐Lorente MA , Cano‐Martin AC , Blasco MA . Mice with hyper‐long telomeres show less metabolic aging and longer lifespans. Nat Commun. 2019;10(1):4723. doi:10.1038/s41467-019-12664-x 31624261 PMC6797762

[10] Bonfigli AR , Spazzafumo L , Prattichizzo F , et al. Leukocyte telomere length and mortality risk in patients with type 2 diabetes. Oncotarget. 2016;7(32):50835‐50844. doi:10.18632/oncotarget.10615 27437767 PMC5239440

[11] Hunt SC , Kark JD , Aviv A . Association between shortened leukocyte telomere length and cardio‐metabolic outcomes. Circ Cardiovasc Genet. 2015;8(1):4‐7. doi:10.1161/CIRCGENETICS.114.000964 25691684

[12] Reddel RR . Telomere maintenance mechanisms in cancer: clinical implications. Curr Pharm Des. 2014;20(41):6361‐6374. doi:10.2174/1381612820666140630101047 24975603 PMC4262939

[13] Schneider CV , Schneider KM , Teumer A , et al. Association of Telomere Length with Risk of disease and mortality. JAMA Intern Med. 2022;182(3):291‐300. doi:10.1001/jamainternmed.2021.7804 35040871 PMC8767489

[14] Qi Nan W , Ling Z , Bing C . The influence of the telomere‐telomerase system on diabetes mellitus and its vascular complications. Expert Opin Ther Targets. 2015;19(6):849‐864. doi:10.1517/14728222.2015.1016500 25677239

[15] Masi S , Gkranias N , Li K , et al. Association between short leukocyte telomere length, endotoxemia, and severe periodontitis in people with diabetes: a cross‐sectional survey. Diabetes Care. 2014;37(4):1140‐1147. doi:10.2337/dc13-2106 24652728

[16] Samavat H , Luu HN , Beckman KB , et al. Leukocyte telomere length, cancer incidence and all‐cause mortality among Chinese adults: Singapore Chinese health study. Int J Cancer. 2021;148(2):352‐362. doi:10.1002/ijc.33211 33459354 PMC10693991

[17] Yeap BB , Hui J , Knuiman MW , et al. U‐shaped relationship of leukocyte telomere length with all‐cause and cancer‐related mortality in older men. J Gerontol A Biol Sci Med Sci. 2021;76(1):164‐171. doi:10.1093/gerona/glaa190 32761187

[18] D'Mello MJ , Ross SA , Briel M , Anand SS , Gerstein H , Pare G . Association between shortened leukocyte telomere length and cardiometabolic outcomes: systematic review and meta‐analysis. Circ Cardiovasc Genet. 2015;8(1):82‐90. doi:10.1161/CIRCGENETICS.113.000485 25406241

[19] Cheng F , Luk AO , Wu H , et al. Shortened relative leukocyte telomere length is associated with all‐cause mortality in type 2 diabetes‐ analysis from the Hong Kong diabetes register. Diabetes Res Clin Pract. 2021;173:108649. doi:10.1016/j.diabres.2021.108649 33422583

[20] Yuan X , Kronstrom M , Hellenius ML , Cederholm T , Xu D , Sjogren P . Longitudinal changes in leukocyte telomere length and mortality in elderly Swedish men. Aging (Albany N Y). 2018;10(10):3005‐3016. doi:10.18632/aging.101611 PMC6224259 30375983

[21] Haycock PC , Heydon EE , Kaptoge S , Butterworth AS , Thompson A , Willeit P . Leucocyte telomere length and risk of cardiovascular disease: systematic review and meta‐analysis. BMJ. 2014;349:g4227. doi:10.1136/bmj.g4227 25006006 PMC4086028

[22] Telomeres Mendelian Randomization C , Haycock PC , Burgess S , et al. Association between telomere length and risk of cancer and non‐neoplastic diseases: a Mendelian Randomization study. *JAMA* . Oncologia. 2017;3(5):636‐651. doi:10.1001/jamaoncol.2016.5945 PMC5638008 28241208

[23] Cheng F , Luk AO , Tam CHT , et al. Shortened relative leukocyte telomere length is associated with prevalent and incident cardiovascular complications in type 2 diabetes: analysis from the Hong Kong diabetes register. Diabetes Care. 2020;43(9):2257‐2265. doi:10.2337/dc20-0028 32661111

